# Fibro‐Adipogenic Progenitors Regulate Orofacial Neuromuscular Junction Regeneration via Myostatin

**DOI:** 10.1002/jcsm.70264

**Published:** 2026-04-01

**Authors:** Ruizhi Li, Ruojing Liu, Yixuan Huang, Yijue Wang, Xu Cheng, Jingtao Li, Shujuan Zou, Xing Yin

**Affiliations:** ^1^ State Key Laboratory of Oral Diseases & National Center for Stomotology & National Clinical Research Center for Oral Diseases & Department of Orthodontics, West China Hospital of Stomatology Sichuan University Chengdu China; ^2^ Department of Stomatology Chengdu Wenjiang District People's Hospital·Wenjiang Hospital of Sichuan Provincial People's Hospital Chengdu China; ^3^ State Key Laboratory of Oral Diseases & National Center for Stomotology & National Clinical Research Center for Oral Diseases & Department of Oral Maxillofacial Surgery, West China Hospital of Stomatology Sichuan University Chengdu China; ^4^ State Key Laboratory of Oral Diseases & National Center for Stomotology & National Clinical Research Center for Oral Diseases & West China School of Stomatology Sichuan University Chengdu China

**Keywords:** acetylcholine receptor, agrin, masseter, mesenchymal stem cells, muscle stem cells

## Abstract

**Background:**

Orofacial and limb muscles differ in embryonic origin and regenerative capacity. Neuromuscular junction (NMJ) regeneration is critical for muscle restoration both histologically and functionally. The relative potential of orofacial and limb muscles to form postsynaptic apparatuses remains elusive. While the role of fibro‐adipogenic progenitors (FAPs) in NMJ regeneration has been discussed in limb muscles, it remains unexplored in orofacial muscles.

**Methods:**

NMJ regeneration was triggered by freeze injury in masseter (MAS) and tibialis anterior (TA) muscles and assessed using histological and functional tests. FAPs transplantation experiments and coculture with muscle stem cells (MuSCs) were performed to investigate their effects on postsynaptic apparatus formation. Transcriptome profiling of FAPs identified the key secretory molecule involved in NMJ regulation. The effect of this molecule was further investigated using in vitro gain‐ and loss‐of‐function assays, conditional knockout transgenic mice and pharmacological blockade.

**Results:**

Immunohistochemistry showed extensive fibrosis surrounded by regenerated myofibres in MAS, whereas no fibrosis but regenerated myofibres in TA. Restored myofibre calibre and resolved fibrosis in the regenerated lesion periphery are observed in both muscles, yet regenerated NMJs remained markedly below the intact level at 30 days post‐injury (dpi) only in MAS (−52.1%, *p* < 0.001). Interestingly, transplantation of FAPs isolated from MAS reduced the number of postsynaptic acetylcholine receptors (AChRs) on regenerated myofibres in recipient TA muscle (−61.3%, *p* < 0.001). Conditioned medium of FAPs isolated from MAS at 7 dpi impaired AChR clustering on myotubes, decreasing the AChR/myotube area ratio (*p* < 0.001). RNA‐seq analysis of 7 dpi MAS and TA FAPs identified myostatin (*Mstn*) as the key differentially expressed gene. *Mstn* transcripts in MAS FAPs were 1.7‐fold higher than those in TA FAPs (*p* < 0.001). In vitro knockdown of *Mstn* in FAPs isolated from 7 dpi MAS reversed its negative effect on AChR clustering, as evidenced by a 4‐fold increase in the AChR/myotube area ratio (*p* < 0.01). The number of nascent AChR clusters in injured MAS of FAP‐specific *Mstn* knockout mice was higher than that of injured floxed controls (2.7‐fold, *p* < 0.001). Pharmacological blockade of MSTN enhanced postsynaptic AChR neogenesis in MAS.

**Conclusions:**

We demonstrated differential NMJ regeneration in MAS and TA muscle. Injury‐activated MAS FAPs impede postsynaptic apparatus formation by secreting pathophysiological levels of MSTN. Lowering MSTN levels in injured MAS might enhance its regeneration through nerve‐muscle signalling.

## 1 Introductions

Orofacial musculature is responsible for an array of critical physiological functions including speech, mastication, deglutition and facial expression. Mastication, primarily executed by the masseter muscle, is actively involved in mandibular morphogenesis [1] and alveolar bone remodelling [2]. Interestingly, orofacial muscles are profoundly different from limb muscles in embryonic origins, developmental patterns, injury responses and disease susceptibilities [3]. Our group previously reported prolonged fibrosis after acute freeze injury in the masseter (MAS) compared with the tibialis anterior (TA) muscle [4].

Neuromuscular junctions (NMJs) are specialized tripartite synapses between motor neurons and muscle fibres. Myotrauma inevitably disturbs NMJ homeostasis. Conversely, NMJ destruction leads to muscle atrophy and dysfunction [5]. Mechanistically, NMJs control muscle movements through neurotransmission and serve as hubs for reciprocal molecular exchanges, maintaining muscle mass and neuron integrity [6]. Therefore, NMJ regeneration is critical to muscle restoration.

Muscle‐derived cytokines (myokines), for example, neurturin [7] and brain‐derived neurotrophic factor (BDNF) [8], are essential in NMJ embryonic formation and postnatal remodelling. In addition, grafting of muscle‐derived fibroblasts and myoblasts into injury sites promotes axon regeneration, possibly via myokine secretion or their multipotent differentiation [9]. In denervated muscle, retaining the pool of Pax7^+^ muscle stem cells (MuSCs) enhanced reinnervation [10]. However, the relative efficiency of NMJ regeneration in orofacial versus limb muscles and the respective roles of muscle‐resident cells remain elusive.

Fibro‐adipogenic progenitors (FAPs) are muscle mesenchymal stem cells characterized by the multipotency to differentiate into fibroblasts, adipocytes, osteocytes and chondrocytes. FAPs exert versatile effects in muscle homeostasis, regeneration and myopathology [11]. A recent work reported that FAPs were adjacent to motor axons and NMJs and preserved NMJ integrity via bone morphogenic protein 3b (BMP3b) secretion [12]. Other studies revealed that FAPs modulated both presynaptic and postsynaptic maturation via survival motor neuron (SMN) expression [13, 14]. FAPs also interact with glial cells in response to nerve injury, either promoting remyelination [15] or inducing fibrosis [16]. Collectively, FAPs play a multifaceted role in NMJ repair. Notably, orofacial FAPs and limb FAPs are derived from cranial neural crest and lateral plate mesoderm, respectively [3, 17]. It remains to be determined whether the above‐mentioned roles of FAPs in limb muscles also apply to orofacial muscles.

In this study, a freeze injury model was used to trigger NMJ regeneration in MAS and TA. The distinct regeneration patterns, cellular players and major molecular mediators were investigated. Our findings demonstrated that FAPs account for the region‐specific NMJ regeneration phenotypes. Further bioinformatic analysis as well as in vivo and in vitro experiments identified FAP‐derived myostatin as the critical suppressor of postsynaptic differentiation.

## 2 Methods

### 2.1 Animals

Male C57BL/6 mice (5‐week‐old) were obtained from Chengdu Dashuo Biological Technology Company, China. PDGFRa^Cre/+^ mice, MSTN^fl/+^ mice and ROSA^nTnG^ mice (C57BL/6 background) were purchased from The Jackson Laboratory. FAP‐specific MSTN KO (MSTN cKO) mice (MSTN^fl/fl^; PDGFRa^Cre/+^) were generated. MSTN^fl/fl^; PDGFRa^+/+^ littermates were used as controls. Three‐week‐old mice received intraperitoneal tamoxifen injection (100 mg/kg) for five consecutive days and were allowed to recover for another 5 days. All mice were housed in a specific pathogen‐free (SPF) facility, maintained at a controlled humidity of 50% ± 5% and temperature of 21°C ± 2°C, with a 12h light/dark cycle. All animal experiments were conducted with three or six mice per group per time point. The experimental protocol was approved by the Institutional Animal Care and Use Committee at Sichuan University (Protocol No. WCHSIRB‐D‐2020‐114) and complied with the ethical standards laid down in the 1964 Declaration of Helsinki and its later amendments.

### 2.2 Freeze Injury Model

Freeze injury was induced in the MAS and TA muscles, following a previously established protocol [4]. Five‐week‐old male C57BL6/J mice (weighing approximately 17 g) were randomly assigned to intact or injury groups with computer‐generated random numbers. Mice were anaesthetised with an intraperitoneal injection of ketamine (100 mg/kg)‐xylazine (5 mg/kg). The MAS and TA muscles were fully exposed following hair removal and skin incision. A piece of dry ice (3.5 mm in diameter and 6–12 mm in length) was held with precooled forceps and applied to the muscles along the long axes for 5 s. The degree of injury was controlled by measuring the recovery time. The site of injury should become stiff and pale upon removing the dry ice and gradually return to its normal texture and colour within 22–25 s. The wound (approximately 7 mm in length) was then closed with 4 stitches using 7‐0 suture. Cages were randomly positioned and rotated weekly. Mice were fed standard chow ad libitum and monitored daily. Animals were excluded if they showed inability to ambulate or feed independently. Mice were euthanized with an overdose of pentobarbital sodium (150 mg/kg, i.p.) at intact state and 14, 30 days post‐injury (dpi). All operations were performed by a single surgeon. Subsequent sample analysis was performed by another investigator blinded to group allocation.

### 2.3 Cell Isolation, Culture and Transfection

For MuSCs isolation, MAS muscles dissected from male C57BL6/J mice (5‐week‐old) were finely minced and digested in 0.1% Pronase (53702, Sigma‐Aldrich, USA) at 37°C for 1 h. The MuSCs were released by two rounds of trituration and then centrifuged at 1000 g for 10 min. The cell pellet was resuspended in growth medium (GM) containing DMEM (4.5 g/L glucose), 20% FBS, 10% HS and 1% P/S. The cells were seeded in Matrigel (356234, Corning, USA) coated plates at a density of 2 × 10^4^ cells/mL. For FAPs isolation, MAS and TA muscles were collected at 7 days post‐injury (7 dpi). A two‐step digestion was performed with collagenase II (LS004176, Worthington, USA) and dispase II (D4693, Sigma‐Aldrich, USA). The cell pellet was resuspended in DMEM with 15% FBS, seeded in T75 flasks and allowed to attach for 3 h at 37°C. The supernatant was discarded and the adherent cells (FAPs) were washed with PBS and supplemented with fresh culture medium. The purity of MuSCs and FAPs was determined by Pax7 and PDGFRα staining. P1 cells were used for future experiments (Figure S2).

To acquire conditioned medium (CM), FAPs from 7 dpi MAS or TA were seeded in T25 flasks and cultured until they reached confluency. The growth medium was then substituted with serum‐free DMEM. After an incubation of 24 h, the CM was collected and spun for 15 min at 3000 g to remove debris. The supernatant was stored in −80°C and thawed on ice before use.

MSTN knockdown was performed in 7 dpi MAS FAPs using pGPU6/GFP/Neo‐shMSTN, while MSTN overexpression was performed in 7 dpi TA FAPs using pcDNA3.1(+)‐MSTN. pGPU6/GFP/Neo‐NC and pcDNA3.1(+) were used as negative controls, respectively. All vectors were generated by GenePharma (Suzhou, China). Transfections were carried out using Lipofectamine 3000 (L3000015, ThermoFisher, USA) according to the manufacturer's instructions. Conditioned medium was acquired 48 h after transfection as aforementioned.

### 2.4 AChR Clustering Assay

MuSCs isolated from the MAS muscles were seeded on Matrigel‐coated (1 mg/mL) coverslips in 24‐well plates at a density of 2 × 10^4^ cells/mL. The GM was replaced with differentiation medium (DM, DMEM + 2% HS + 1% P/S) at a confluency of 70%. Mature myotubes were treated with 100 ng/mL agrin (HY‐P79236, MedChemExpress, China) in DM or conditioned medium (CM) of 7 dpi FAPs supplemented with 2% HS for 16 h. For recombinant MSTN treatment, 100 ng/mL MSTN (HY‐P72632, MedChemExpress, China) with or without 500 ng/mL follistatin (HY‐P70315, MedChemExpress, China) was administered together with agrin treatment. For *miR‐206* transfection, *miR‐206* mimic and negative control (NC) were designed and synthesized by Genecarer (Xi'an, China). Transfected myotubes were subsequently treated with agrin to induce acetylcholine receptor (AChR) clustering, as described above.

### 2.5 FAPs Transplantation

Male C57BL/6 mice (5‐week‐old) were used as recipients and ROSA^nTnG^ mice were used as donors. Recipient mice were freeze‐injured at either MAS or TA muscle. After 24 h, 6 × 10^4^ FAPs (suspended in 50 μl PBS) isolated from donor mice were injected intramuscularly into the injured sites. Donor FAPs were marked by tdTomato fluorescence. Specifically, the injured MAS or TA muscle received tdTomato^+^ FAPs isolated from either the MAS or TA muscle of donor mice. An injection of 50 ul PBS was used as controls.

### 2.6 ACE‐031 Administration

Male C57BL/6 mice (5‐week‐old) were randomly assigned to intact, PBS or ACE‐031 treatment groups (*n* = 3 per group). ACE‐031 (10 mg/kg, Peptidego, China) was injected subcutaneously at 1 dpi, 7 dpi and 14 dpi. An equivalent volume of PBS was administered to PBS controls.

### 2.7 Quantitative Real‐Time PCR

RNA was extracted from cultured cells using TRIzol (Invitrogen, USA) according to the manufacturer's protocol. RNA was reverse transcribed into cDNA using the PrimeScript FAST RT reagent Kit with gDNA Eraser (Takara, Japan). Quantitative real‐time PCR was performed using the TB Green Premix Ex Taq II (Tli RNaseH Plus, Takara, Japan) on a LightCycler480 system (Roche Diagnostics, USA). Reactions were performed in triplicate and the gene expression levels were normalized to *Gapdh* using the 2^−ΔΔCt^ method. All primers used were listed in Table S1.

For qRT‐PCR detection of *miR‐206*, total RNA was first treated with DNase I (RNase‐free) (Takara, Japan) to remove any contaminating DNA fragments. The RNA was then polyadenylated and reverse transcribed into cDNA, followed by quantification by qRT‐PCR using the Mir‐X miRNA qRT‐PCR TB Green Kit (Takara, Japan). The relative gene expression was normalized to *U6*. The forward primer (5′‐GGTGGAATGTAAGGAAGTGTGTGG‐3′) was used, and the reverse primer was supplied with the kit.

### 2.8 RNA Sequencing and Analysis

FAPs were isolated from the MAS and TA muscle at 7 dpi. Total RNA was extracted using the RNA‐easy Isolation Reagent (R701‐01, Vazyme, China) and assessed for quality. Libraries were generated and sequenced. Data cleaning was performed and clean reads were aligned to the reference genome. Finally, differential expression analysis was conducted. We compiled a list of NMJ‐regulating genes from the AmiGO 2 database and relevant literature, then intersected the DEGs with this list and filtered for those encoding secreted proteins.

### 2.9 Enzyme‐Linked Immunosorbent Assay

Concentration of MSTN in FAPs CM was detected by ELISA following the manufacturer's instructions (Human/Mouse/Rat GDF‐8 ELISA Kit, KE00120, Proteintech, China). FAPs were cultured in 24‐well plates (4 × 10^4^ cells/mL) until confluence before serum‐free DMEM was added for 24 h. The CM was collected, centrifuged and subjected to ELISA. Optical density at 405 nm was determined with a microplate reader (Tecan, Switzerland).

### 2.10 Western Blotting Analysis

FAPs were lysed with RIPA (PC101, Epizyme Biotech, China) on ice. Protein concentration was determined using a BCA protein assay kit (ZJ101, Epizyme Biotech, China). Equal amounts of protein extracts were separated in a 10% SDS‐PAGE and transferred to PVDF membrane (Millipore, USA). After blocking, the membranes were probed with primary antibodies overnight at 4°C. HRP‐conjugated secondary antibodies were incubated for 1 h at RT the following day. Images were acquired using a ChemiDoc system (Bio‐Rad, USA). Semiquantitative analyses were performed using ImageJ2 (NIH). The primary antibodies used were MSTN antibody (ab203076, Abcam, USA) and GAPDH antibody (14C10, Cell Signaling, USA).

### 2.11 Tissue Immunofluorescence and Histology

Intact and injured MAS and TA muscles were harvested, embedded in OCT and flash‐frozen in liquid N2‐cooled isopentane. Muscles were cryosectioned at 500 μm intervals and 30 μm longitudinal or 10 μm transverse sections of the middle region were collected. Sections were fixed with ice‐cold acetone for 15 min, blocked and permeabilized with 5% BSA, 5% goat serum and 0.1% Triton X‐100 in PBS. After incubation with primary antibodies overnight at 4°C, secondary antibodies were applied at RT for 1 h. The following primary antibodies were used: neurofilament 200 (1:100, N4142, Sigma‐Aldrich, USA) and laminin (1:500, L9393, Sigma‐Aldrich, USA). Secondary antibodies include: Goat Anti‐Rabbit DyLight 488 (1:200, Abbkine, China). The postsynaptic AChRs were labelled with TRITC‐α‐bungarotoxin (BTX) (1:500, Biotium, USA) or 488‐α‐BTX (1:500, Biotium, USA). The sections were cover‐slipped with the Antifade Mounting Medium with DAPI (Beyotime, China). Images were taken at 40× objective with the FV3000 Olympus confocal microscope (Olympus Corporation, Tokyo, Japan). Z‐stack images were analysed with ImageJ (NIH). Innervation was defined by the overlap of presynaptic and postsynaptic signals.

For histological analysis, cryosections were stained with the haematoxylin–eosin (H&E) stain kit or Modified Sirius Red Stain Kit (Solarbio, China). Images were taken with the VS200 fluorescence microscope (Olympus Corporation, Tokyo, Japan) and analysed with ImageJ (NIH).

### 2.12 Immunocytochemistry

Cells were fixed in 4% PFA for 15 min, rinsed with PBS and then blocked with 2% BSA and permeabilised with 0.1% Triton X‐100 in PBS for 30 min at RT. Coverslips were incubated overnight at 4°C with primary antibodies. Subsequently, cells were incubated with secondary antibodies and TRITC‐α‐BTX (1:500, Biotime, China) for 1 h at RT. Primary antibodies include MyHC antibody (1:20, MF20, DSHB, USA), MyoG antibody (1:20, F5D, DSHB, USA), Pax7 antibody (1:10, PAX7, DSHB, USA) and PDGFRα antibody (1:300, AF1062, R&D Systems, USA). Secondary antibodies include Goat Anti‐Mouse DyLight 488 (1:200, Abbkine, China) and IFKine^TM^ Red Donkey anti‐Goat (1:200, Abbkine, China). Coverslips were mounted on glass slides with the antifade mounting medium with DAPI (Beyotime, China) for later observation. Z‐stack images were taken with FV3000 Olympus Confocal microscope at 20× and analysed with ImageJ2 (NIH). AChR clusters were defined by an area of > 3 μm^2^. AChR cluster area was normalized to myotube area. Differentiation index was calculated by the number of nuclei in MyHC+ cells divided by the total number of nuclei.

### 2.13 Motor Function Analysis

Bite force and hind‐limb grip time were recorded at intact state and at 30 dpi. Bite force was measured using the FlexiForce B201 sensor and FlexiForce ELF System (Tekscan, USA). Mice were acclimated for 30 min in a custom‐made clear plastic cage with a small opening. The sensor was first calibrated linearly by a series of weight loads (1–1000 g). Each mouse was tested 5 times with a duration of 1 min and an interval of 3 min. For hindlimb grip time measurements, mice were acclimated on the mesh lid for 30 min. The mouse was brought close to the mesh lid, letting it grasp the wire with only hindlimbs. The mouse was gently released and the fall latency was timed. Each mouse was timed 3 times, with an interval of 30 min.

### 2.14 Statistical Analyses

All data are presented as mean ± SD and were analysed using GraphPad Prism (version 10.0, GraphPad Software Inc., USA). Data normality was evaluated using the Shapiro–Wilk test. Unpaired Student's *t* tests were used for two‐group comparisons. One‐ or two‐way ANOVA followed by Tukey's post hoc test or Sidak post hoc test were used for multiple‐group comparisons. Nonparametric equivalents were applied when data did not conform to normality or homoscedasticity. *p* < 0.05 was considered statistically significant.

## 3 Results

### 3.1 MAS Exhibits Retarded NMJ Regeneration Compared With TA

**FIGURE 1:**
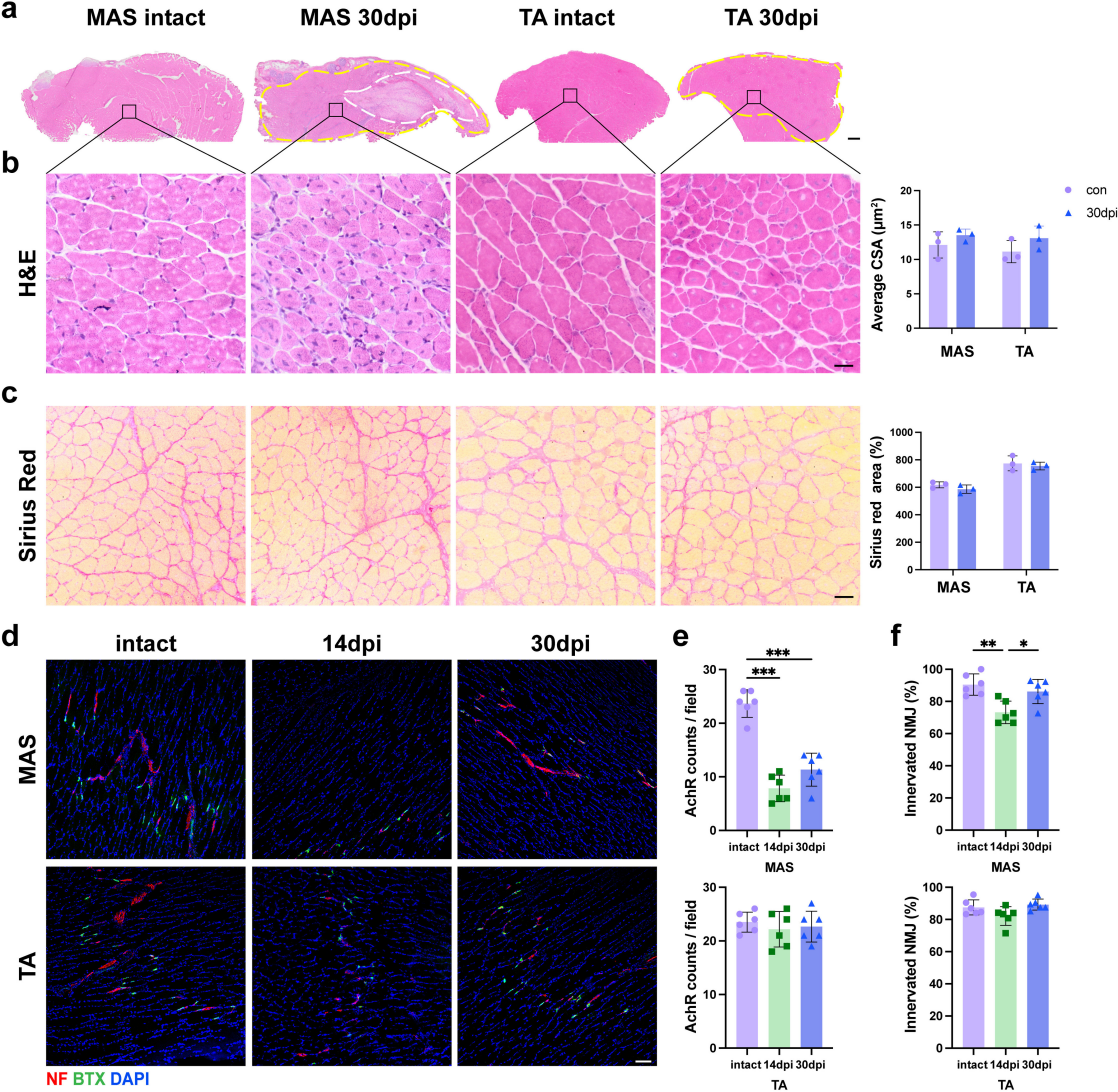
Inferior NMJ regeneration in MAS compared with TA. (a) Overview of the MAS and TA cross‐section. A prominent fibrotic lesion was seen in MAS at 30 dpi (White dashed line). Yellow dashed line indicated injured sites marked by centrally nucleated myofibres. Scale bar = 200 μm. (b) Representative images of H&E staining at the lesion periphery and quantification of the mean cross‐sectional area. Scale bar = 20 μm. *n* = 3 mice/group. (c) Representative images of sirius red staining at the lesion periphery at different time points and quantification of the fibrotic area. Scale bar = 20 μm. *n* = 3 mice/group. (d) Immunofluorescent staining for NMJs of the longitudinal sections. Representative images showing neurofilament (NF) (red) and AChRs labelled with α‐BTX (green). Scale bar = 100 μm (e) The number of AChR clusters per field in intact and injured MAS (upper) and TA (lower). *n* = 6 mice/group. (f) The percentage of innervated NMJs in intact and injured MAS (upper) and TA (lower). *n* = 6 mice/group. The data are shown as mean ± SD. Two‐way ANOVA followed by Sidak post hoc test was used in (b,c). One‐way ANOVA followed by Tukey's post hoc test was used in (e,f). ns, not significant, *p < 0.05, **p < 0.01.

By 30 dpi, TA exhibited complete regeneration as evidenced by centrally nucleated myofibres. Although MAS showed extensive fibrotic scarring at the injury centre, regenerated myofibres of intact size were present at the lesion periphery (Figure 1a,b). Likewise, collagen deposition quantified by sirius red staining returned to intact levels in both muscles in this area (Figure 1c). Thus, the lesion periphery was used for subsequent NMJ analyses. At 14 dpi, the number of nascent AChR clusters in TA reached the level of intact controls, while that in MAS remained significantly lower than control even at 30 dpi (Figure 1d,e). The percentage of innervated NMJs was comparable to intact levels in both MAS and TA at 30 dpi (Figure 1f). Functional assessments revealed incomplete recovery of masticatory force but full recovery of hindlimb grip time at 30 dpi (Figure S1a,b). Taken together, the functional impairment of MAS might be attributed to a combination of prolonged fibrosis in the lesion centre and insufficient NMJ regeneration in the lesion periphery.

### 3.2 FAPs Account for the Disparity in NMJ Regeneration Between MAS and TA

**FIGURE 2:**
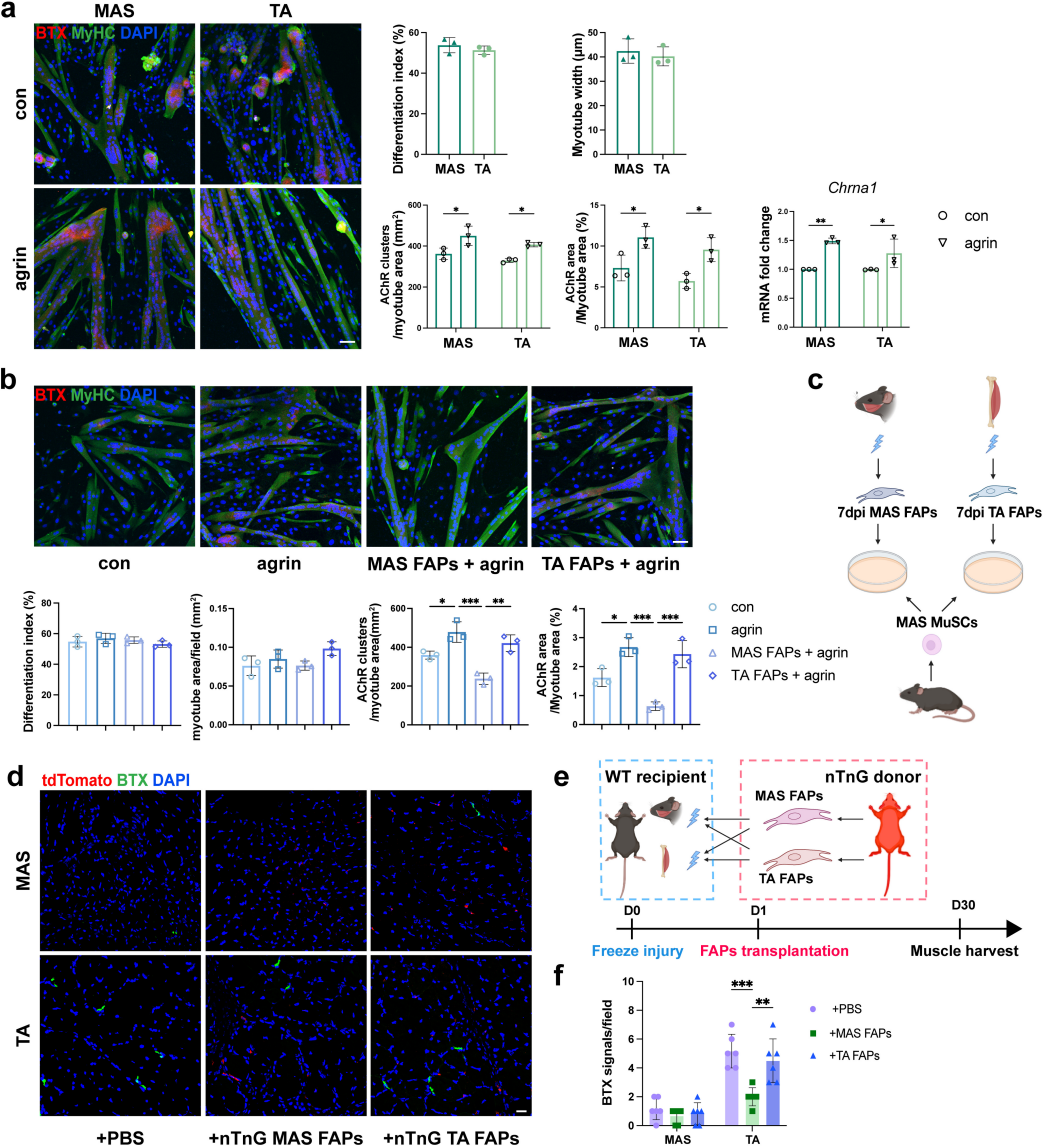
FAPs accounted for the disparity in NMJ regeneration between MAS and TA muscles. (a) AChR and MyHC immunolabelling in myotubes differentiated from MuSCs isolated from MAS or TA muscles. Mature myotubes were treated with or without agrin (100 ng/mL) for 16 h. Scale bar = 50 μm. Graphs in the right panel show the quantifications of differentiation index (upper left), myotube width (upper right), AChR cluster counts normalized to myotube area (lower left), ratio of AChR/myotube area (lower middle) and *Chrna1* expression quantified by real‐time quantitative PCR (lower right). *n* = 3. (b) AChR and MyHC staining of MuSCs single culture and cocultures with different FAPs. Scale bar = 50 μm. Graphs in the lower panel show (from left to right) the quantification of differentiation index, myotube area, AChR cluster density and ratio of AChR/myotube area. *n* = 3. (c) Schematic of the direct coculture of different FAPs with MAS‐derived MuSCs. (d) Representative images of tdTomato^+^ FAPs and α‐BTX labelled AChRs (green) in recipient MAS and TA muscle. Scale bar = 20 μm. (e) Schematic of the FAPs transplantation assay. (f) Quantification of BTX signals per field. *n* = 6 mice/group. The data are shown as mean ± SD. Unpaired Student's *t* test was used in (a, upper right panel). Two‐way ANOVA followed by Tukey's post hoc test was used in (a, lower right panel and f). One‐way ANOVA followed by Tukey's post hoc test was used in (b). ns, not significant, *p < 0.05, **p < 0.01, ***p < 0.001.

To investigate whether the insufficient postsynaptic regeneration in MAS reflects an intrinsic deficit in orofacial MuSCs, in vitro myogenic differentiation and AChR clustering assays were performed. MAS‐ and TA‐derived MuSCs did not differ significantly in myogenin (MyoG) expression (Figure S2b), differentiation index, or myotube diameter (Figure 2a). Myotubes differentiated from both cells exhibited similar AChR clustering in response to agrin stimulation, which was further confirmed by the AChR subunit gene *Chrna1* transcription (Figure 2a). Thus, the impaired AChR clustering in MAS was more likely the result of cues extrinsic to MuSCs.

FAPs undergo dynamic transcriptional changes upon injury and are known to modulate MuSCs behaviour during regeneration [11]. In this light, FAPs isolated from MAS and TA muscles at 7 dpi were cocultured with MAS‐derived MuSCs (Figure 2c). While the differentiation index and myotube area remained unchanged, coculture with MAS FAPs markedly reduced both AChR cluster density and the AChR/myotube area ratio (Figure 2b). To further exclude confounding influences of the resident muscle milieu, we conducted a cross transplantation experiment (Figure 2e). Engraftment of MAS or TA FAPs did not alter the already low level of BTX signals in MAS. However, the transplanted MAS FAPs hampered NMJ regeneration in TA, while TA FAPs made no significant difference (Figure 2d,f). Collectively, FAPs of distinct origins differentially influence postsynaptic AChR formation on myotubes both in vivo and in vitro.

### 3.3 Elevated MSTN Secretion Distinguishes MAS‐Derived FAPs From TA‐Derived Ones

**FIGURE 3:**
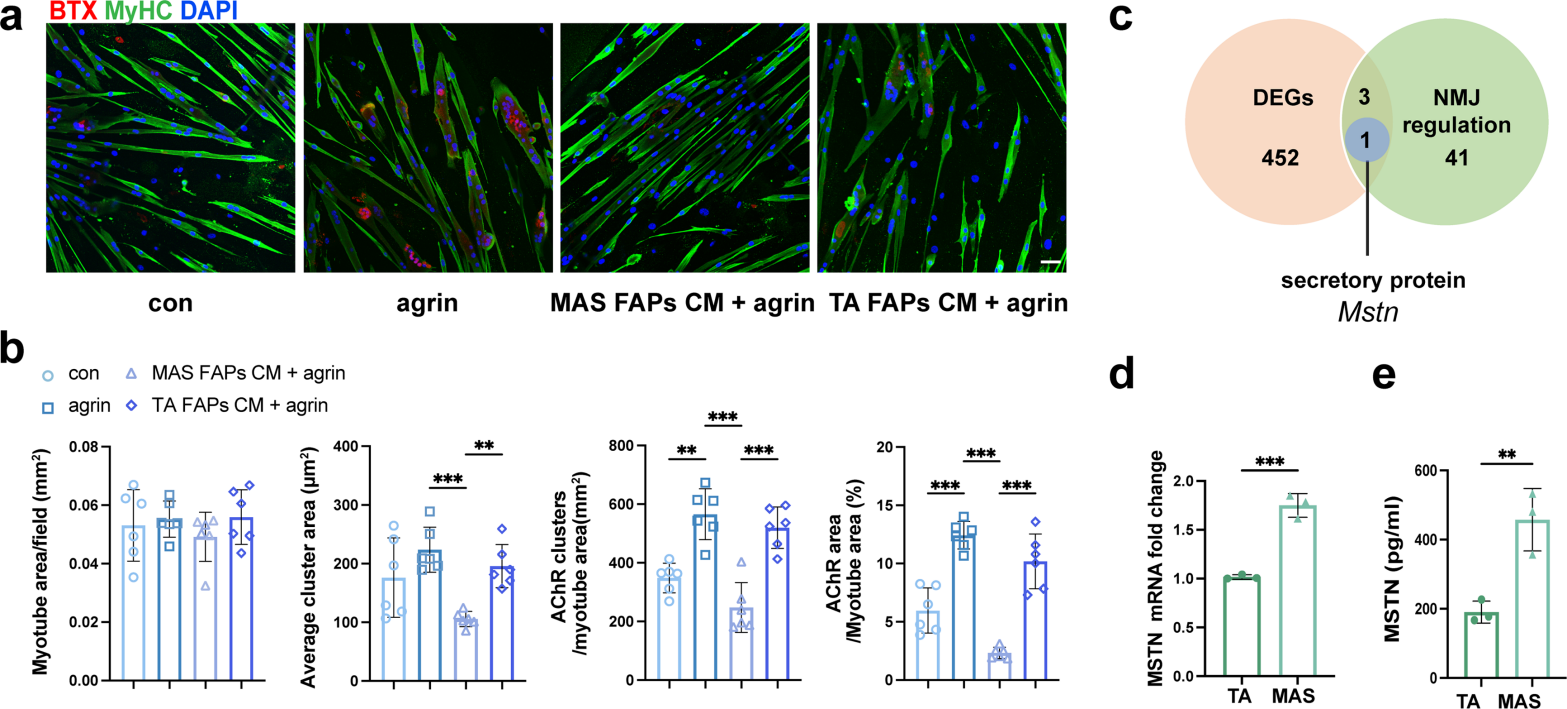
MSTN was the paracrine factor that distinguishes MAS‐derived FAPs from TA‐derived FAPs. (a) AChR and MyHC staining of MuSC single culture or cocultures with conditioned medium (CM) of MAS‐ or TA‐isolated FAPs. Scale bar = 50 μm. (b) Graphs show (from left to right) the quantification of myotube area, average AChR cluster size, AChR cluster counts normalized to myotube area and ratio of AChR/myotube area. *n* = 6. (c) Venn diagram depicting the overlap among DEGs between 7 dpi MAS‐ and TA‐derived FAPs (orange), NMJ‐regulating genes (green) and genes encoding secreted proteins (blue). (d) *Mstn* expression levels were quantified by qPCR. *n* = 3. (e) Myostatin levels were quantified by ELISA in CM from 7 dpi MAS‐ or TA‐derived FAPs. *n* = 3. The data are shown as mean ± SD. One‐way ANOVA followed by Tukey's post hoc test was used in (b). Unpaired Student's *t* test was used in (d,e). ns, not significant, *p < 0.05, **p < 0.01, ***p < 0.001.

Next, we explored whether FAPs regulate AChR clustering through juxtacrine or paracrine mechanisms. The conditioned medium (CM) from MAS or TA 7 dpi‐FAPs was added to mature myotubes differentiated from MAS‐derived MuSCs. MAS FAPs CM lowered AChR cluster density, cluster size and AChR/myotube area fraction (Figure 3a,b), indicating that MAS FAPs secrete factors that inhibit AChR clustering. Bulk RNA‐seq identified 452 differentially expressed genes, of which 3 were implicated in NMJ regulation. Among them, *Mstn* encodes a secretory protein, myostatin (MSTN), a negative regulator of muscle growth and regeneration [18] (Figure 3c). Elevated *Mstn* expression in MAS FAPs was confirmed by qPCR (Figure 3d), and higher MSTN concentration was detected in the CM of MAS FAPs (Figure 3e).

### 3.4 Recombinant MSTN Inhibits AChR Clustering and *miR‐206* Expression

**FIGURE 4:**
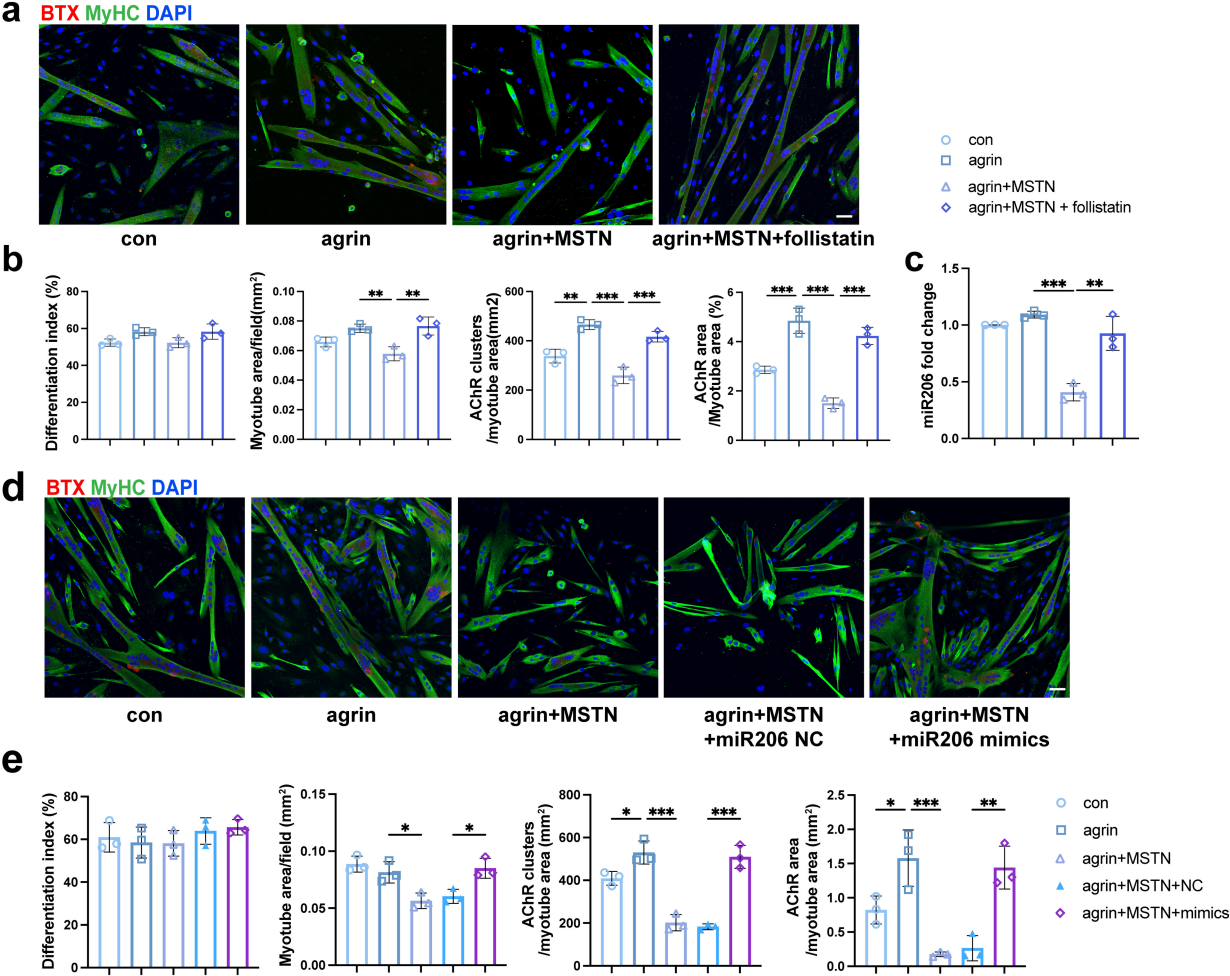
Recombinant MSTN suppressed AChR clustering and *miR‐206* expression. (a) AChR and MyHC staining of MuSCs cultures treated with or without recombinant MSTN (100 ng/mL) and follistatin (500 ng/mL). Scale bar = 50μm. (b) Graphs show (from left to right) the quantification of differentiation index, myotube area, AChR cluster counts normalized to myotube area and ratio of AChR/myotube area. *n* = 3. (c) The levels of *miR‐206* expression in MuSCs cultures. Mature myotubes differentiated from MAS‐derived MuSCs were treated with control, agrin alone, agrin plus MSTN or agrin plus MSTN and follistatin. (d) AChR and MyHC staining of myotubes transfected with *miR‐206* mimics or negative control (NC) prior to treatment with agrin and MSTN. Scale bar = 50 μm. (e) Graphs show (from left to right) the quantification of differentiation index, myotube area, AChR cluster counts normalized to myotube area and ratio of AChR/myotube area. *n* = 3. The data are shown as mean ± SD. One‐way ANOVA followed by Tukey's post hoc test was used. ns, not significant, *p < 0.05, **p < 0.01, ***p < 0.001.

To elucidate whether MSTN impairs AChR clustering, recombinant MSTN was applied with agrin to mature myotubes. MSTN treatment led to decreased AChR density and area fraction, which was reversed by the MSTN antagonist follistatin [18] (Figure 4a,b). *MiR‐206*, a muscle‐specific microRNA, has previously been implicated in AChR clustering [19]. MSTN treatment lowered *miR‐206* levels in myotubes, and this reduction was reversed by follistatin (Figure 4c). Transfection of *miR‐206* mimics rescued the MSTN‐suppressed AChR clustering (Figure 4d,e), suggesting that *miR‐206* may act downstream of MSTN.

### 3.5 MSTN Knockdown in MAS‐Derived FAPs Reversed Their Negative Effect on AChR Clustering

**FIGURE 5:**
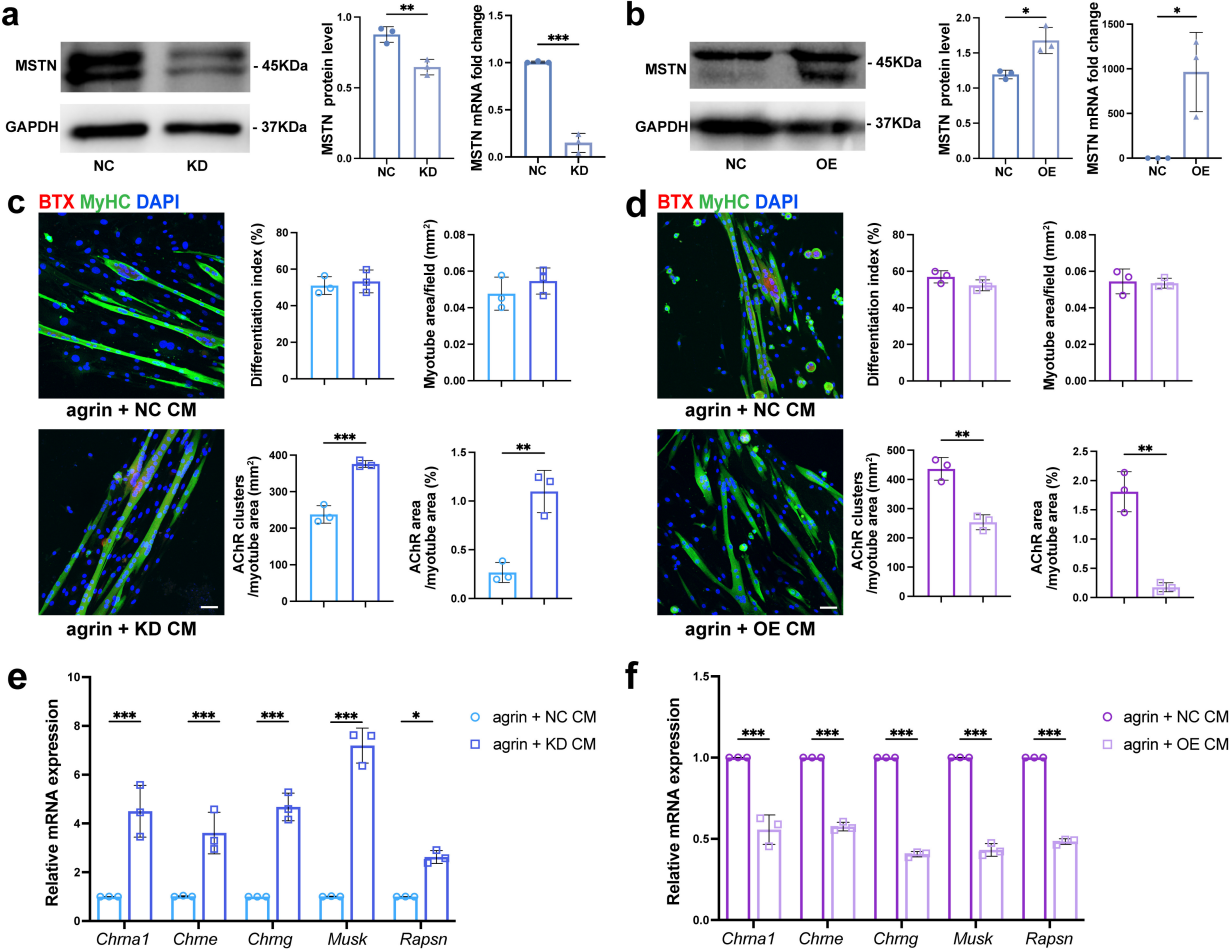
MSTN knockdown reversed the negative effect of MAS FAPs on in vitro AChR clustering. (a) MSTN protein and mRNA level in 7 dpi MAS FAPs transfected with negative control (pGPU6/GFP/Neo‐NC) or knockdown vectors (pGPU6/GFP/Neo‐shMSTN). (b) MSTN protein and mRNA level in 7 dpi TA FAPs transfected with negative control (pcDNA3.1(+)) or overexpression vectors (pcDNA3.1(+)‐MSTN). (c) Representative images of AChR and MyHC staining (left panel). Graphs in the right panel show the quantification of differentiation index (upper left), myotube area (upper right), AChR cluster counts normalized to myotube area (lower left) and ratio of AChR/myotube area (lower right). CM was collected from NC and MSTN KD FAPs and added to myotubes differentiated from MAS‐derived MuSCs. Scale bar = 50 μm. *n* = 3. (d) Representative images of AChR and MyHC staining (left panel). Graphs in the right panel show the quantification of differentiation index (upper left), myotube area (upper right), AChR cluster density (lower left) and ratio of AChR/myotube area (lower right). CM was collected from NC and MSTN OE FAPs and added to myotubes differentiated from MAS‐derived MuSCs. Scale bar = 50 μm. *n* = 3. (e,f) Transcript levels of *Chrna1*, *Chrne*, *Chrng*, *Musk* and *Rapsn* in myotubes treated with CM from NC or KD FAPs, and NC or OE FAPs. KD, knock down; OE, overexpression. The data are shown as mean ± SD. Unpaired Student's *t* test was used in (a–d). Two‐way ANOVA followed by Sidak post hoc test was used in (e,f). ns, not significant, *p < 0.05, **p < 0.01, ***p < 0.001.

Additionally, MSTN knockdown in 7 dpi MAS FAPs and overexpression in 7 dpi TA FAPs were performed. WB and qPCR results confirmed the efficiency of gene manipulation (Figure 5a,b). CM of MSTN‐KD FAPs, MSTN‐OE FAPs and their corresponding controls was collected, supplemented with agrin and applied to MAS MuSCs‐derived myotubes. Increased AChR density and area ratio were observed in myotubes treated with MSTN‐KD CM than the NC group (Figure 5c). Conversely, treatment with MSTN‐OE CM reduced AChR density and area ratio relative to control CM (Figure 5d). Concordant changes were seen in transcripts encoding AChR subunits (*Chrna1*, *Chrne*, *Chrng*) and clustering regulators (*Musk*, *Rapsn*) (Figure 5e,f). Thus, MSTN levels underlie the contrasting roles of MAS and TA FAPs, and altering MSTN expression is sufficient to reverse their effects on AChR clustering in vitro.

### 3.6 Systemic or FAPs‐Specific MSTN Blockade Enhances NMJ Regeneration in MAS

**FIGURE 6:**
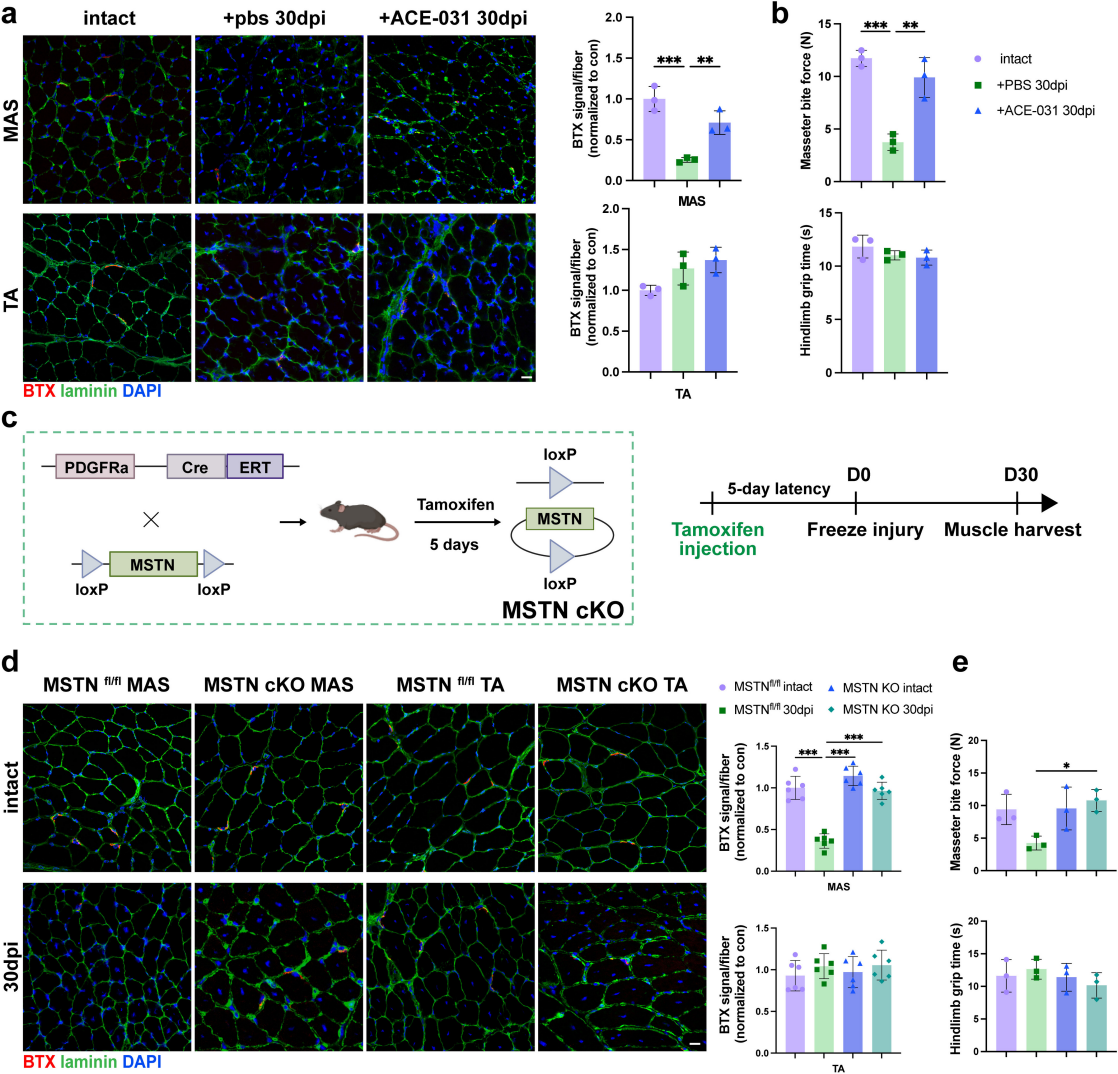
FAP‐specific MSTN knockout and pharmacological blockade of MSTN enhanced NMJ regeneration in MAS muscle. (a) AChR immunostaining of transverse sections from intact, PBS‐treated, or ACE‐031‐treated mice. Graphs in the right panel show the quantification of AChR counts per myofibre in MAS (upper) and TA (lower). *n* = 3 mice/group. (b) Masseter bite force in intact, PBS‐treated and ACE‐031‐treated mice at 30 dpi (upper). Hindlimb grip time assessed in intact, PBS‐treated and ACE‐031‐treated mice at 30 dpi (lower). *n* = 3 mice/group. (c) Schematic showing the generation of FAPs‐specific MSTN knockout mice (MSTN cKO). (d) AChR labelling of transverse sections of MAS or TA muscles of MSTN cKO mice (MSTN^fl/fl^; Pdgfra^Cre/+^) and control mice (MSTN^fl/fl^; Pdgfra^+/+^). Graphs in the right panel show the quantification of AChR counts per myofibre in MAS (upper) and TA (lower). *n* = 6 mice/group. (e) Masseter bite force (upper) and hindlimb grip time (lower) were measured in intact and injured MSTN cKO mice and controls. *n* = 3 mice/group. The data are shown as mean ± SD. One‐way ANOVA followed by Tukey's post hoc test was used. ns, not significant, *p < 0.05, **p < 0.01, ***p < 0.001.

Intraperitoneal injection of ACE‐031, a decoy receptor of MSTN [18], increased the number of regenerated AChR clusters in MAS as compared with PBS injection controls (Figure 6a). Functionally, ACE‐031 treatment markedly restored masticatory forces. No significant effect of ACE‐031 was noticed in TA (Figure 6b).

FAP‐specific MSTN KO mice (MSTN cKO) were generated and assessed for NMJ regeneration (Figure 6c). In accordance with WT phenotypes, MSTN‐floxed controls showed scarce AChR signals per myofibre in regenerating MAS at 30 dpi, far below the intact level. Notably, MSTN cKO markedly elevated AChR signals in injured MAS. Meanwhile, AChR regeneration remained robust in TA of either MSTN cKO or control mice (Figure 6d). Similarly, masticatory function was substantially recovered in MSTN cKO (Figure 6e). Collectively, either systemic MSTN blockade or FAP‐specific MSTN knockout suffices to improve NMJ regeneration in MAS.

## 4 Discussion

Our present study pioneeringly reported the region‐specific role of FAPs in NMJ regeneration. We first evaluated NMJ regeneration disparities in MAS and TA after freeze injury. Markedly less de novo NMJs were found in MAS despite considerable myofibre regeneration in the lesion periphery. Further in vitro experiments and FAPs transplantation assay confirmed the regulatory role of FAPs in postsynaptic apparatus regeneration. Mechanistically, FAPs in MAS expressed more MSTN in response to acute injury, which inhibited AChR clustering in myotubes via *miR‐206* down‐regulation. FAP‐specific *Mstn* knockout reversed the phenotype. Finally, pharmacological blockade of MSTN improved NMJ regeneration in MAS. Collectively, our results demonstrated distinctive roles of different FAPs in postsynaptic differentiation, validating FAP‐targeting strategies to enhance orofacial muscle repair.

As the interface between nerve and muscle, the NMJ is vulnerable to damage in either compartment. On one hand, nerve injury results in myofibre atrophy, interstitial fibrosis and devascularization [20]. On the other hand, myotrauma, including laceration [21], volumetric muscle loss [22], and ischaemia–reperfusion [23], inevitably disrupts NMJ integrity, leading to reduced synapse counts, AChR fragmentation and denervation. Yet NMJ disruption following freeze injury, another widely employed injury model, remained uncharacterized. Freeze injury differs fundamentally from other myotoxin‐ or chemical‐induced injuries. A ‘dead zone’ devoid of viable cells and a regenerative front of infiltrating myoblasts were induced, making it an ideal model for muscle regeneration [24]. We reported insufficient NMJ regeneration in MAS compared with TA at 30 dpi, a deficit consistent with the pronounced fibrotic phenotype previously reported in MAS [4]. Distinctive embryonic origins and motor unit distribution may underlie the disparate regeneration. Craniofacial muscles derive from cranial mesoderm, while limb muscles derive from the somites [3]. A motor unit is a motor neuron and all the myofibres it innervates. Fibres from different motor units are interspersed in a mosaic pattern. Data from rabbit and cat (comparable in size) suggests denser motor unit fibre distribution in the masseter muscle than limb muscles, despite comparable fibres per motor unit [25].

During ΝΜJ development, aneural AChR clusters form at the middle region of muscles prior to nerve ingrowth. Subsequently, agrin released from nerve terminals stabilizes these pre‐patterned clusters or induces new ones. Mechanistically, agrin binds to the LRP4/MuSK co‐receptor, which phosphorylates a series of downstream proteins, ultimately leading to AChR anchoring by Rapsyn [26]. Our study reported comparable myogenic and AChR clustering capacities between MuSCs isolated from MAS and TA. However, a previous study had reported more active proliferation and reluctant differentiation of MAS‐derived MuSCs compared with those from limb muscles [27]. These seemingly contradictory results may stem from different culturing methods of the previous research.

Our cross‐transplantation experiment suggested that FAPs of different origins might shape the microenvironment they reside in. The effect of FAPs on NMJ homeostasis is supported by a growing body of studies. Recent snRNA sequencing of WT FAPs identified a subpopulation surrounding AChRs that expand in response to nerve transection [28]. Depletion of PDGFRα^+^ FAPs resulted in similar NMJ deteriorations observed in sarcopenia [12]. FAP‐specific overexpression of *Bmp3b* rescued age‐related NMJ degeneration [29]. The Ret/Gfra1^+^ FAPs secrete BDNF in response to Schwann cell‐derived GDNF, promoting remyelination and axon repair [15]. Additionally, FAPs play a dual role in peripheral nerve repair. Activated FAPs initially enhance NMJ regeneration through neurogenic signalling with glial cells, while later depositing fibrillar ECM that impedes nerve regeneration [16].

FAPs are recognized to influence myogenesis via paracrine factors, as demonstrated in a series of coculture experiments with MuSCs. FAP‐derived WISP1 promotes MuSC expansion and myogenic commitment. WISP1 expression is elevated in FAPs upon cardiotoxin (CTX) or glycerol induced injury [30]. Compared with MuSCs, FAPs are the main sources of secretory follistatin, which promotes myoblast fusion into myotubes [31]. Wildtype FAPs promote myotube hypertrophy by soluble BMP3b via Akt and Smad1/5/8 signalling [12]. However, the involvement of FAPs in AChR clustering in myotubes has not been investigated. We reported that in vitro co‐culturing with FAPs derived from injured TA had no significant effect on AChR clustering in myotubes differentiated from MAS‐derived MuSCs. In contrast, FAPs derived from injured MAS impaired the process.

Myostatin (MSTN) is a highly conserved negative regulator of muscle growth, involved in multiple steps of myogenesis. MSTN suppresses the proliferation and differentiation of MuSCs by inhibiting cell cycle progression and downregulating myogenic regulatory factors such as myogenin, myoblast determination protein 1 (MyoD), and myogenic factor 5 (Myf5). MSTN also induces myotube atrophy by downregulating protein synthesis via inhibition of the IGF‐1/PI3K/Akt/mTOR pathway and upregulating ubiquitin ligases such as muscle atrophy F‐box (MAFbx)/Atrogin‐1 and muscle RING finger 1 (MuRF‐1) through FOXO1 activation [18]. Apart from influencing the lineage progression of myogenic cells, MSTN and its orthologues are also involved in neurogenesis. In Drosophila, myoglianin (MYO, the orthologue of myostatin) inhibits the formation of both presynaptic and postsynaptic components at larval NMJs. Specifically, muscle‐specific downregulation of MYO increased the number of presynaptic active zones and postsynaptic GluRIIB glutamate receptors [32]. In zebrafish, *mstnb*, the orthologue of the mammalian *MSTN* gene, is highly enriched in muscle tissue. It promotes the self‐renewal of neurogenic progenitors via fibroblast growth factor 1 (FGF1). Conversely, *mstnb* loss of function not only enhanced skeletal muscle regeneration after spinal cord injury but also promoted neural differentiation of Sox2^+^ progenitors in the dorsal spinal cord [33]. MSTN knockout leads to increased number of total axons and motor axons innervating the TA muscle [34]. Building on previous studies, we reported the negative influence of supraphysiological levels of MSTN on AChR formation in myotubes. To rule out the atrophic effects of MSTN on myotubes, we normalized the AChR area to the myotube area.

MicroRNAs are small noncoding RNAs associated with post‐transcriptional regulations of mRNA. *MiR‐206*, a member of the muscle‐specific microRNAs, is closely related to MuSCs differentiation, muscle mass regulation and neuromuscular disease progression [35]. The gene clusters *miR‐1/206/133* combined modulate AChR cluster formation and maintenance via regulation of the CRK‐RAC1 cascade activity [36]. Transit activation of RAC1 initiates AChR clustering, while constant activation disperses pre‐existing clusters [37]. In a healthy state, *miR‐1/206/133* limits RAC1 activation via CRK repression, ensuring proper AChR clustering. Triple knockout of *miR‐1/206/133* disrupts this balance and compromises NMJ formation and maintenance [36]. Another study reported that overexpression of *miR‐206* reduced the number and size of AChR clusters by downregulating Rapsyn and other proteins involved in AChR anchoring [19]. Taken together, physiological levels of *miR‐206* are essential for NMJ homeostasis. We reported decreased *miR‐206* expression in myotubes upon exogenous MSTN treatment. This is consistent with the previous finding that MSTN knockout upregulated *miR‐206* levels [38].

We proposed that MSTN represses AChR clustering via microRNA regulation. However, the underlying mechanisms remain to be elucidated. Additionally, it should be noted that other mechanisms may mediate the effects of FAPs on NMJ regeneration. For example, FAPs are potent sources of ECM components, including those of the synaptic basal lamina (SBL) [39]. Therefore, FAPs may influence NMJ regeneration via ECM modulation. Leveraging the RNA‐seq dataset, we plan to explore FAP‐mediated SBL regulation in follow‐up works. Furthermore, it remains to be validated whether disparate FAPs dynamics and MSTN levels are conserved across injury types, calling for future investigation of MSTN blockade in other clinically relevant muscle injury models.

In conclusion, MAS and TA exhibit divergent NMJ regeneration after freeze injury, a disparity attributable to higher MSTN expression in injured MAS‐derived FAPs. MSTN may be a potential therapeutic target to enhance NMJ regeneration in orofacial muscle.

## Funding

This work was supported by the National Natural Science Foundation of China (82271017), Sichuan Science and Technology Program (2025ZNSFSC0769), Sichuan Medicine Youth Innovative Research Project (Q23038), Align Technology Research Program (No. AQKY22‐2‐6) and Natural Science Foundation of Sichuan Province (2023NSFSC0034).

## Conflicts of Interest

The authors declare no conflicts of interest.

## Supporting information


**Table S1:** Primer Sequences.

**Figure S1:** Incomplete functional recovery of the masseter muscle. (a) Bite force measured in intact mice and at 30 dpi. (b) Hind‐limb grip time assessed in intact mice and at 30 dpi. *n* = 3 mice/group. The data are shown as mean ± SD ns, not significant, *p ≤ 0.05, **p ≤ 0.01.

**Figure S2:** Validation of MuSCs and FAPs cell culture. (a) Pax7 staining of MuSCs isolated from MAS and TA. Scale bar = 20 μm. (b) MyoG staining of differentiating MuSCs isolated from MAS and TA. Scale bar = 50 μm. Quantification of the percentage of MyoG+ nuclei. *n* = 3. (c) PDGFRα staining of FAPs isolated from 7dpi MAS and TA. Scale bar = 50 μm. The data are shown as mean ± SD ns, not significant.

## Data Availability

The datasets used and/or analysed in this study are available from the corresponding author upon reasonable request.
