# Supplementary material for: Fibro‐Adipogenic Progenitors Regulate Orofacial Neuromuscular Junction Regeneration via Myostatin
Source: J Cachexia Sarcopenia Muscle. 2026 Apr 1;17(2):e70264. doi: 10.1002/jcsm.70264 (PMC13045380; doi:10.1002/jcsm.70264)
Supplement: Supplementary file 1 — Table S1: Primer Sequences. [file JCSM-17-e70264-s002.docx]

**FAPs Regulate Orofacial Neuromuscular Junction Regeneration via Myostatin**

Ruizhi Li^1^, Ruojing Liu^1,3^, Yixuan Huang^2^, Yijue Wang^4^, Xu Cheng^2^, Jingtao Li^2^, Shujuan Zou^1^, Xing Yin^1,*^

1. State Key Laboratory of Oral Diseases & National Clinical Research Center for Oral Diseases & Dept. of Orthodontics, West China Hospital of Stomatology, Sichuan University, Chengdu, China
2. State Key Laboratory of Oral Diseases & National Clinical Research Center for Oral Diseases & Dept. of Oral Maxillofacial Surgery, West China Hospital of Stomatology, Sichuan University, Chengdu, China
3. Dept. of stomatology, Chengdu Wenjiang District People’s Hospital·Wenjiang Hospital of Sichuan Provincial People’s Hospital, Chengdu, China
4. State Key Laboratory of Oral Diseases & National Clinical Research Center for Oral Diseases & West China School of Stomatology, Sichuan University, Chengdu, China

* Corresponding author: X. Yin, State Key Laboratory of Oral Diseases & National Clinical Research Center for Oral Diseases & Dept. of Orthodontics, West China Hospital of Stomatology, Sichuan University, No. 14, Third Section, Renmin South Road, Chengdu 610041, Sichuan, China.

Email: yinxing@scu.edu.cn

**Table S1. Primer Sequences.**

| Target Gene | Forward (5'-3') | Reverse (3'-5') |
| --- | --- | --- |
| *Chrna1* | CCACAGACTCAGGGGAGAAG | AACGGTGGTGTGTGTTGATG |
| *Chrne* | GTGTCTGGATTGGCATTGACT | ACACCTGCAAAATCGTCCTTG |
| *Chrng* | GAGAGCCACCTCGAAGACAC | GACCAACCTCATCTCCCTGA |
| *Musk* | CTTCAGCGGGACTGAGAAAC | TGTCTTCCACGCTCAGAATG |
| *Rapsn* | TGGGGCAGGACCAGACAAAGCAAC | TCCAGTCCCCGAGCAGTATCAATC |
| *Mstn* | AGTGGATCTAAATGAGGGCAGT | GTTTCCAGGCGCAGCTTAC |
| *Gapdh* | AGGTCGGTGTGAACGGATTTG | TGTAGACCATGTAGTTGAGGTCA |
| *U6* | GGAACGATACAGAGAAGATTAGC | TGGAACGCTTCACGAATTTGCG |

**Figure S1.** Incomplete functional recovery of the masseter muscle. (a) Bite force measured in intact mice and at 30 dpi. (b) Hind-limb grip time assessed in intact mice and at 30 dpi. n

= 3 mice/group. The data are shown as mean ± SD. ns, not significant, *p ≤ 0.05, **p ≤ 0.01.

**Figure S2.** Validation of MuSCs and FAPs cell culture. (a) Pax7 staining of MuSCs isolated from MAS and TA. Scale bar = 20 μm. (b) MyoG staining of differentiating MuSCs isolated from MAS and TA. Scale bar = 50 μm. Quantification of the percentage of MyoG+ nuclei. n = 3. (c) PDGFRα staining of FAPs isolated from 7dpi MAS and TA. Scale bar = 50 μm.The data are shown as mean ± SD. ns, not significant.
