# Supplementary figures and images for: Fibro‐Adipogenic Progenitors Regulate Orofacial Neuromuscular Junction Regeneration via Myostatin
Source: J Cachexia Sarcopenia Muscle. 2026 Apr 1;17(2):e70264. doi: 10.1002/jcsm.70264 (PMC13045380; doi:10.1002/jcsm.70264)

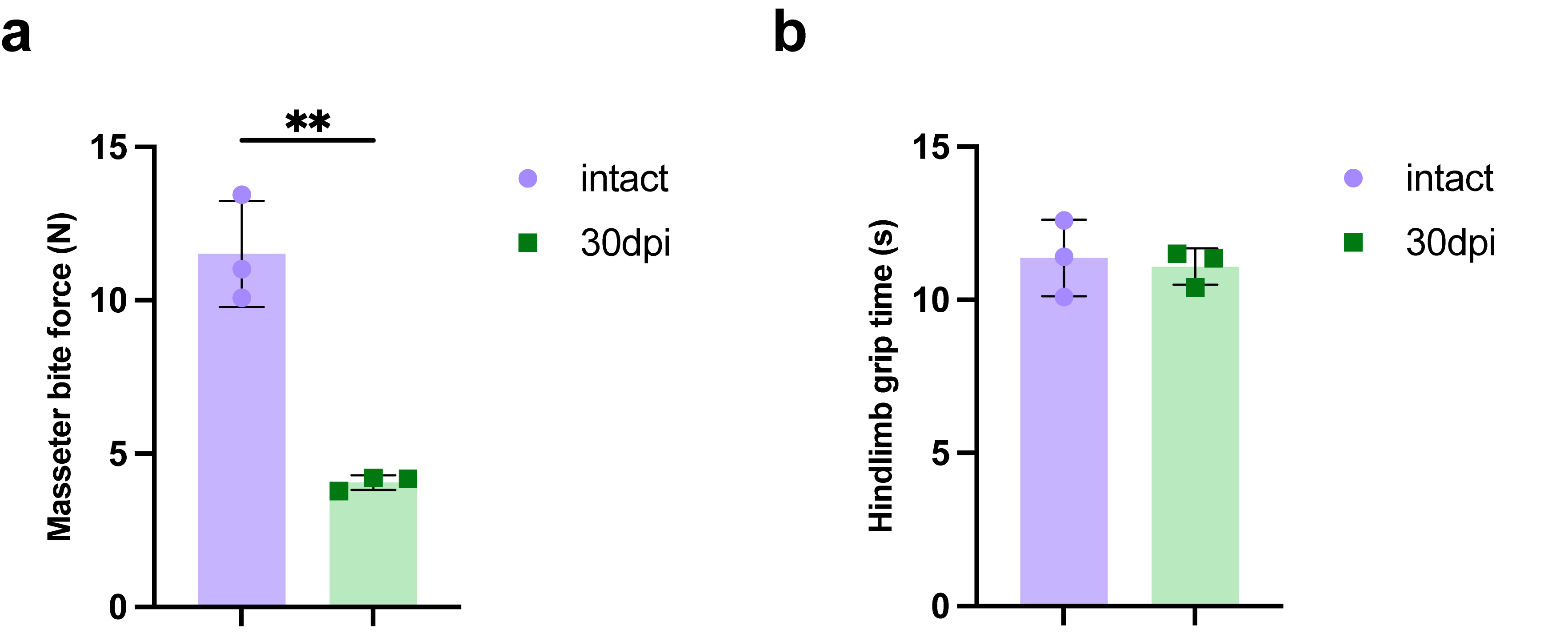

Supplement: Supplementary file 2 — Figure S1: Incomplete functional recovery of the masseter muscle. (a) Bite force measured in intact mice and at 30 dpi. (b) Hind‐limb grip time assessed in intact mice and at 30 dpi. n = 3 mice/group. The data are shown as mean ± SD ns, not significant, *p ≤ 0.05, **p ≤ 0.01. [file JCSM-17-e70264-s001.tif]

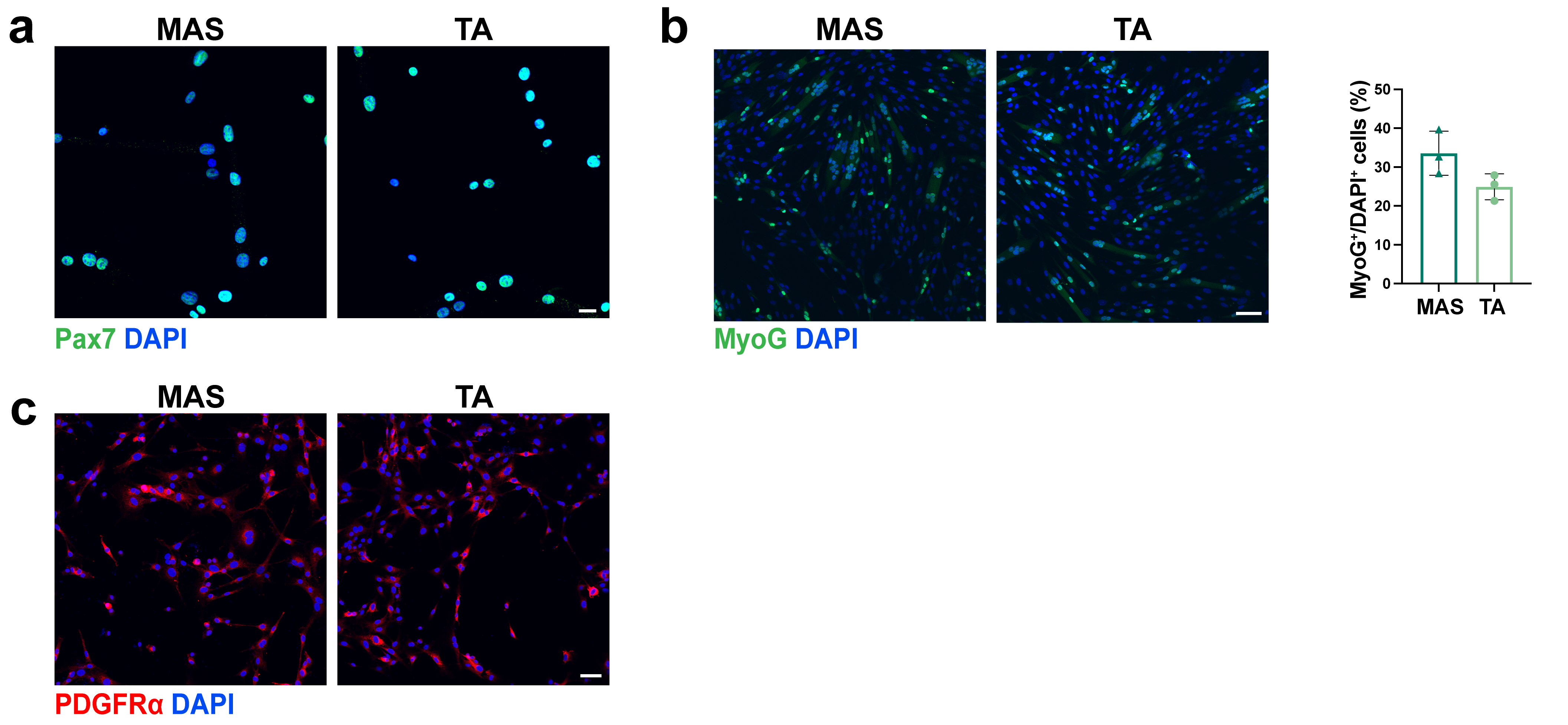

Supplement: Supplementary file 3 — Figure S2: Validation of MuSCs and FAPs cell culture. (a) Pax7 staining of MuSCs isolated from MAS and TA. Scale bar = 20 μm. (b) MyoG staining of differentiating MuSCs isolated from MAS and TA. Scale bar = 50 μm. Quantification of the percentage of MyoG+ nuclei. n = 3. (c) PDGFRα staining of FAPs isolated from 7dpi MAS and TA. Scale bar = 50 μm. The data are shown as mean ± SD ns, not significant. [file JCSM-17-e70264-s003.tif]
